# Mortality in the Year Following Antiretroviral Therapy Initiation in HIV-Infected Adults and Children in Uganda and Zimbabwe

**DOI:** 10.1093/cid/cis797

**Published:** 2012-09-12

**Authors:** A. Sarah Walker, Andrew J. Prendergast, Peter Mugyenyi, Paula Munderi, James Hakim, Addy Kekitiinwa, Elly Katabira, Charles F. Gilks, Cissy Kityo, Patricia Nahirya-Ntege, Kusum Nathoo, Diana M. Gibb, for the DART and ARROW trial teams

**Affiliations:** 1 MRC Clinical Trials Unit, London; 2 Queen Mary University of London, United Kingdom; 3 Joint Clinical Research Centre, Kampala; 4 MRC/UVRI Uganda Research Unit on AIDS, Entebbe; 5 University of Zimbabwe Clinical Research Centre, Harare; 6 Baylor-Uganda Pediatric Infectious Disease Centre, Mulago; 7 Infectious Disease Institute, Makarere University, Kampala, Uganda; 8 Imperial College, London, United Kingdom; 9 University of Zimbabwe Medical School, Harare

## Abstract

***Background.*** Adult mortality in the first 3 months on antiretroviral therapy (ART) is higher in low-income than in high-income countries, with more similar mortality after 6 months. However, the specific patterns of changing risk and causes of death have rarely been investigated in adults, nor compared with children in low-income countries.

***Methods.*** We used flexible parametric hazard models to investigate how mortality risks varied over the first year on ART in human immunodeficiency virus–infected adults (aged 18–73 years) and children (aged 4 months to 15 years) in 2 trials in Zimbabwe and Uganda.

***Results.*** One hundred seventy-nine of 3316 (5.4%) adults and 39 of 1199 (3.3%) children died; half of adult/pediatric deaths occurred in the first 3 months. Mortality variation over year 1 was similar; at all CD4 counts/CD4%, mortality risk was greatest between days 30 and 50, declined rapidly to day 180, then declined more slowly. One-year mortality after initiating ART with 0–49, 50–99 or ≥100 CD4 cells/μL was 9.4%, 4.5%, and 2.9%, respectively, in adults, and 10.1%, 4.4%, and 1.3%, respectively, in children aged 4–15 years. Mortality in children aged 4 months to 3 years initiating ART in equivalent CD4% strata was also similar (0%–4%: 9.1%; 5%–9%: 4.5%; ≥10%: 2.8%). Only 10 of 179 (6%) adult deaths and 1 of 39 (3%) child deaths were probably medication-related. The most common cause of death was septicemia/meningitis in adults (20%, median 76 days) and children (36%, median 79 days); pneumonia also commonly caused child deaths (28%, median 41 days).

***Conclusions.*** Children ≥4 years and adults with low CD4 values have remarkably similar, and high, mortality risks in the first 3 months after ART initiation in low-income countries, similar to cohorts of untreated individuals. Bacterial infections are a major cause of death in both adults and children; targeted interventions could have important benefits.

## Abstract (precis)

In low-income countries, children ≥4 years and adults with low CD4 count have equally high mortality risk in the 3 months after initiation of antiretroviral therapy, similar to that of untreated individuals. Bacterial infections play a major role; targeted interventions could have important benefits.

By December 2010, 6.65 million human immunodeficiency virus (HIV)–infected adults and children in low- and middle-income countries were receiving antiretroviral therapy (ART) [1]; however, this was only 47% of those in need. Adults in these settings have an excess mortality risk during the first 2–3 months on ART compared with those in high-income settings, even after adjusting for important cofactors (including CD4 count, age, sex, and ART regimen), with more-similar mortality risks thereafter [2]. Although early mortality has declined in low-income settings over the last decade, this has mainly been driven by fewer severely immunocompromised individuals starting ART [3–5]; for example, in 7 South African programs [4], 12-month mortality declined from 9% to 6% during 2002–2007 as the median pre-ART CD4 count increased from 69 to 113 cells/μL. Nevertheless, early mortality remained approximately 6-fold higher in those initiating ART with CD4 <50 cells/μL vs >200 cells/μL even in the latest period [4].

Although 2010 World Health Organization (WHO) guidelines recommend ART at a CD4 count threshold of <350 cells/μL [6], patients continue to present with low CD4 counts or to refuse ART at higher CD4 counts [7]. If only 15% of the 7.5 million untreated HIV-infected persons in need [1] have CD4 <50 cells/μL and experience 7% early mortality, this corresponds to 78 750 deaths in this subgroup alone. However, few data inform how excess early mortality might be reduced [8]. While several adult studies have demonstrated substantial declines in mortality risk on ART [2, 9–13], time has been variably and arbitrarily categorized [14], preventing precise exploration of how mortality risk changes following ART initiation. Furthermore, few studies have addressed this question in children, and only 1 study has compared mortality risks with those of adults [15]. We therefore investigated how mortality risk changed over time in adults and children starting ART, using flexible statistical models for these risks, and explored possible reasons that might inform rational interventions. We used pooled data from 2 large African adult and pediatric trials of ART management strategies, in which mortality ascertainment was near complete.

## METHODS

Mortality during the first year on ART was estimated in HIV-infected adults (aged 18–73 years) and children (aged 4 months to 15 years) enrolled in the Development of Antiretroviral Therapy in Africa (DART) [16] and Antiretroviral Research for Watoto (ARROW; www.arrowtrial.org) trials, respectively. Both trials recruited previously untreated individuals (except to prevent mother-to-child-transmission) from 3 centers in Uganda and 1 in Zimbabwe. In DART, adults had a CD4 count <200 cells/μL and symptomatic (WHO stage 2/3/4) HIV disease; children in ARROW met WHO 2006 pediatric guidelines for ART [17] due to immunosuppression (age <12 months: CD4% <25%; age 1 to <3 years: <20%; age 3 to <5 years: <15% [or CD4 <350 cells/μL]; age ≥5 years: <15% [or CD4 <200 cells/μL]) and/or WHO 3/4 stage disease.

The primary randomized comparison in both trials was clinically driven monitoring (CDM) vs routine laboratory plus clinical monitoring (LCM) for toxicity (hematology/biochemistry) and efficacy (CD4 counts); no real-time HIV loads were assayed. As there was no evidence of mortality or disease progression differences between randomized groups during the first 2 years in DART [16], and no plausible mechanism for early differences in mortality other than toxicity-related deaths (see Results below), groups were pooled for analysis of 12-month mortality on ART; subsequent deaths and follow-up were censored at this time point. At enrollment in 2003–2004, DART participants received 3-drug ART (coformulated zidovudine/lamivudine plus tenofovir, abacavir, or nevirapine), open-label except for 600 participants randomized to abacavir vs nevirapine [18]. In 2007–2008, ARROW participants received open-label 3- or 4-drug ART (abacavir/lamivudine plus a nonnucleoside reverse transcriptase inhibitor [NNRTI] or zidovudine + NNRTI); participants starting 4 drugs reduced to 3 drugs at 36 weeks.

Participants in both studies saw a doctor and underwent full blood count, lymphocyte subset (CD4, CD8), and biochemistry testing (bilirubin, urea, creatinine, alanine aminotransferase, and aspartate aminotransferase) every 12 weeks; they were reviewed by a nurse using a standard symptom checklist every 4 weeks. All LCM results were returned to clinicians, whereas CDM results were only returned if requested for clinical reasons (not CD4) or grade 4 laboratory toxicity. For all participants, diagnostic investigations and other tests (except CD4/lymphocytes for CDM) could be requested, concomitant medications prescribed, and antiretrovirals substituted for adverse events. Switching for “failure” before 48 weeks was discouraged [17, 19]. In both trials, participants missing visits were contacted by phone or by fieldworker visit. All reported WHO stage 4 events and deaths were reviewed by an endpoint review committee (ERC) with independent chair and members, who assigned cause of death blinded to randomization.

To estimate a continuously varying death rate (hazard), we used flexible parametric models [20, 21] counting time from ART initiation to earliest of death, loss to follow-up, or 1 year (see Supplementary Methods). Pre-ART CD4 was categorized by absolute CD4 counts in participants aged ≥4 years and CD4 cell percentage (CD4%) in those aged <4 years, given similar predictive ability of CD4 in untreated children aged ≥4 years and adults [22, 23]. Prespecified categories were 0–49 cells/μL or 0%–4%; 50–99 cells/μL or 5%–9%; 100–149 cells/μL or 10%–14%; and ≥150 cells/μL or ≥15%. The impact of CD4/CD4%, age, sex, WHO stage, and cotrimoxazole at ART initiation was investigated in multivariable models. A second multivariable model also included pre-ART hemoglobin and body mass index (BMI) converted into BMI-for-age *z* scores following WHO guidelines [24, 25]. Norms at 19 years were used for adults (*z* score = 0 at BMI = 22.2 [men] and 21.4 [women]). Incidence of different causes of death were compared between adults and children using cause-specific hazards (competing risk subhazards [26] were similar, data not shown). Exact tests were used where no child deaths meant these could not be estimated. All analyses were performed using Stata software version 11.2.

## RESULTS

A total of 3316 adults (aged 18–73 years) and 1206 children (aged 4 months to 17 years) initiated ART in DART and ARROW, respectively, in the same Zimbabwean/Ugandan centers. As there were few older adolescents, participants aged 16–17 years (n = 7) were excluded from analysis. More adults than children had severe immunodeficiency (Table 1), likely reflecting earlier accrual (2003–2004 vs 2007–2008) and more stringent DART eligibility criteria of CD4 <200 cells/μL and symptomatic HIV disease. Of 428 children aged 4–15 years with CD4 ≥200 cells/μL, 46% had <350 cells/μL and 48% had a CD4% of <15%, reflecting some discrepancies between CD4/CD4% in children [23]; remaining children with neither (36%) initiated ART for WHO 3/4 events.

**Table 1.** Characteristics at Antiretroviral Therapy Initiation

Factor	DART (N = 3316)	ARROW 4–15 y (n = 738)	ARROW 0–3 y (n = 461)
Center			
Entebbe, Uganda	1020 (31%)	135 (18%)	52 (11%)
IDI/PIDC, Uganda	300 (9%)	194 (26%)	118 (26%)
JCRC, Uganda	997 (30%)	286 (39%)	114 (25%)
Harare, Zimbabwe	999 (30%)	123 (17%)	177 (38%)
Women/girls	2156 (65%)	369 (50%)	236 (51%)
Age, y	36 (31–42)	8 (6–10)	1 (1–2)
Pre-ART CD4, cells/μL	86 (31–139)	251 (95–398)	725 (471–1081)
0–49 (0%–4% if <4 y)	1109 (33%)	131 (18%)	27 (6%)
50–99 (5%–9% if <4 y)	785 (24%)	56 (8%)	87 (19%)
100–149 (10%–14% if <4 y)	759 (23%)	52 (7%)	128 (28%)
150–199 (15%–19% if <4 y)	663 (20%)	71 (10%)	98 (21%)
≥200 (≥20% if <4 y)	0^a^	428 (58%)	121 (26%)
WHO stage			
1/2	673 (20%)	214 (29%)	136 (30%)
3	1864 (56%)	444 (60%)	235 (51%)
4	779 (23%)	80 (11%)	90 (20%)
Weight, kg	57 (50–64)	20 (17–26)	9 (7–11)
BMI, kg/m^2^	21.1 (19.1–23.6)	15.0 (14.0–15.9)	15.6 (14.2–16.7)
BMI-for-age *z* score (WHO^b^)	−0.2 (−1.0 to 0.6)	−0.8 (−1.5 to −0.1)	−0.2 (−1.6 to 0.7)
Hemoglobin, g/dL	11.4 (10.3–12.7)	11.1 (10.2–11.9)	9.9 (9.2–10.7)
On cotrimoxazole prophylaxis	2048^c^ (62%)	735^c^ (99.6%)	460^d^ (99.8%)
First ART regimen			
ZDV/3TC/TDF	2469 (74%)	0	0
ZDV/3TC/ABC	300 (9%)	0	0
ZDV/3TC/NVP	547 (16%)	0	0
3TC/ABC/EFV	0	128 (17%)	11^e^ (2%)
3TC/ABC/NVP	0	111 (15%)	143 (31%)
ZDV/3TC/ABC/EFV	0	284 (38%)	19^e^ (4%)
ZDV/3TC/ABC/NV	0	215 (29%)	288 (62%)

Data are presented as No. (%) or median (interquartile range). Weight and BMI not available pre-ART for 23 and 33 DART participants, respectively.

Abbreviations: 3TC, lamivudine; ABC, abacavir; ARROW, Antiretroviral Research for Watoto; ART, antiretroviral therapy; BMI, body mass index; DART, Development of Antiretroviral Therapy in Africa; EFV, efavirenz; IDI/PIDC, Infectious Diseases Institute/Pediatric Infectious Diseases Clinic (Mulago Hospital); JCRC, Joint Clinical Research Center; NVP, nevirapine; TDF, tenofovir; WHO, World Health Organization; ZDV, zidovudine.

^a^ All DART participants had CD4 <200 cells/μL at ART initiation as trial entry criterion.

^b^ For adults, calculated using WHO references [24], assuming age 19 years.

^c^ In addition, 3 (0.1%) adults and 3 (0.4%) older children were taking dapsone prophylaxis at ART initiation.

^d^ One child had grade 2 neutropenia at ARROW enrollment and initiated cotrimoxazole 4 months later.

^e^ No dosing available for EFV in children <3 years of age or <15 kg.

Thirty-eight (1.1%) adults and 9 (0.8%) children had unknown vital status at 1 year. In contrast to many programs [27], pre-ART CD4 counts were similar between those lost to follow-up and those followed up at 1 year (adults: median 117 vs 88 cells/μL, *P* = .26 [vs 33 cells/μL among deaths in year 1]; children: 501 vs 368 cells/μL, *P* = .30 [vs 103 cells/μL among deaths in year 1]). Participants lost to follow-up were therefore censored at last clinic attendance (median 26 weeks [adults] and 20 weeks [children]). Eleven (0.3%) adults and 3 (0.3%) children switched to second-line ART (all but 1 adult >46 weeks).

**Figure 1.**
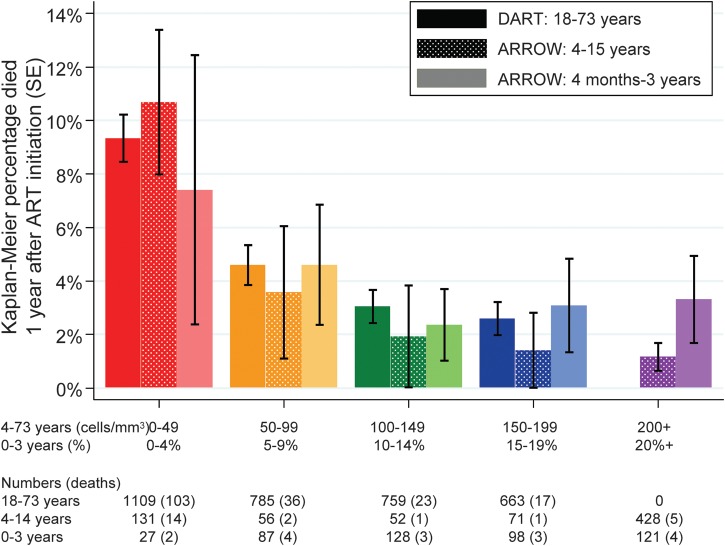
Kaplan-Meier mortality 1 year after antiretroviral therapy (ART) initiation according to age and pre-ART CD4 count. Abbreviations: ART, antiretroviral therapy; ARROW, Antiretroviral Research for Watoto; DART, Development of Antiretroviral Therapy in Africa.

Overall, 179 (5.4%) adults and 39 (3.3%) children died in the first year following initiation of ART. As expected, mortality varied strongly with pre-ART CD4 (*P *< .0001 for adults and older children), with no statistical evidence of variation in younger children (*P* = .70). Interestingly, mortality risk was similar in adults and older children (≥4 years) in the same CD4 strata (Figure 1). Risk was also similar in younger children (<4 years) in parallel CD4% strata, although power was low to detect genuine differences. Results were similar with a 5-year threshold [19] and in older children with CD4 <350 cells/μL (data not shown). Risk gradients were much stronger <100 cells/μL (or <10% for age <4 years); above this, differences were smaller with no evidence supporting important variation (*P *> .6). Subsequent analyses therefore pooled CD4 >100 cells/μL.

**Figure 2.**
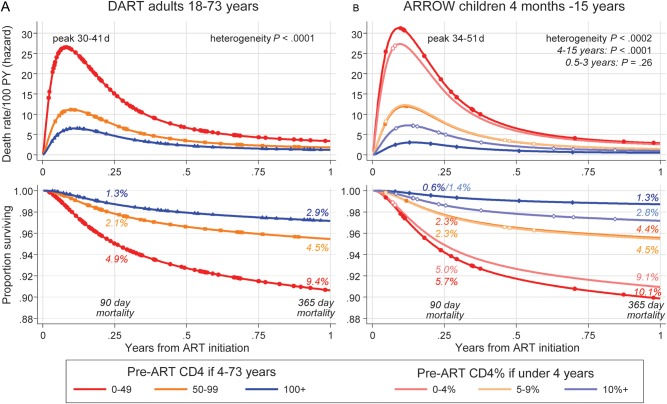
Daily risk of death and survival through 1 year on antiretroviral therapy (ART) according to age and pre-ART CD4 count. Flexible parametric model [20, 21] on log-normal scale with 1 interior knot. Points show times when deaths occurred. Abbreviations: ART, antiretroviral therapy; ARROW, Antiretroviral Research for Watoto; DART, Development of Antiretroviral Therapy in Africa; PY, person-years.

Figure 2 shows how mortality risk varies day-by-day over the first year on ART. At all CD4/CD4%, and in adults and children, mortality risk increased from enrollment to a maximum between 30 and 50 days after ART initiation, declined rapidly to 180 days, then declined more slowly. In both adults and children, half and three-quarters of the deaths occurred in the first 3 and 6 months, respectively. The sharp initial risk increase is likely because of trial consent (excluding moribund patients); the earliest deaths occurred on day 8 (DART) and day 16 (ARROW). In sensitivity analyses, assuming the 1% of participants lost to follow-up had died, differences between groups were similar. Pooling data from adults and children, there was no evidence for a differential effect of pre-ART CD4 with age (heterogeneity *P* = .95 [0–49 cells/μL, 0%–4%]; *P* = .98 [50–99 cells/μL, 5%–9%]; and *P* = .15 [≥100 cells/μL, ≥10%]).

Adjusting for CD4/CD4%, mortality risks were lower in those with earlier pre-ART WHO stage or on cotrimoxazole at ART initiation (*P* < .0001), but there was no evidence of additional effects of age, sex, or center (*P* > .16; Supplementary Table 1). After adjusting for increased mortality risk with lower pre-ART hemoglobin and BMI (*P* < .0001), there was weak evidence of slightly increased mortality risk in older individuals on ART (*P* = .02).

There was no evidence supporting a large contribution of ART (or other drug) toxicity to mortality on ART during the first 3 months or the first year (Table 2). Only 6% of adult and 3% of child year-1 deaths were ERC-adjudicated as primarily medication-related (4% and 3% primarily ART-related, respectively; Table 2). ART-related deaths were from septicemia (4 adults, 1 child), neutropenia without sepsis (2 adults), anemia (1 adult), and hepatitis (1 adult). Sixty percent of primarily medication-related deaths occurred in those with pre-ART CD4 <50 cells/μL, similar to deaths that were uncertainly medication-related (62%) and primarily HIV-related (68%).

**Table 2.** Causes of Death in the First Year on Antiretroviral Therapy

					Median (IQR) d From ART Initiation to Death		Deaths Within 3 mo of ART Initiation	
	DART 18–73 y (n = 179)	ARROW 4 mo to15 y (n = 39)	Crude Cause-Specific (ARROW:DART) HR (95% CI), *P* Value	Adjusted^a^ Cause-Specific (ARROW:DART) HR (95% CI), *P* Value	DART^b^	ARROW^b^	DART (n = 90)	ARROW (n = 20)
Relationship to HIV and drugs								
Primarily HIV related	92 (51%)	28 (72%)	.83 (.54–1.27), .39	1.34 (.86–2.09), .19	69 (32–160)	86 (42–153)	50 (56%)	15 (75%)
Primarily medication related	10^c^ (6%)	1^d^ (3%)	.27 (.04–2.14), .22	.43 (.05–3.54), .44	53 (25–75)	39 (29–47)	9 (10%)	0
Uncertain whether primarily HIV or medication related	24^e^ (13%)	5^f^ (13%)	.57 (.22–1.49), .25	.91 (.34–2.47), .85	81(47–128)	…	13 (14%)	5 (25%)
Uncertain whether HIV related or not, but not medication related	1 (1%)	1 (3%)	…	…	…	…	1 (1%)	0
Uncertain whether medication related or not, but not HIV related	1^e^(1%)	0	…	…	…	…	0	0
Unlikely to be HIV or medication related	17 (9%)	1 (3%)	.16 (.02–1.20), .08	.16 (.02–1.26), .08	141 (86–216)	…	6 (7%)	0
Relationship to HIV/medications could not be determined	34 (19%)	3 (8%)	.24 (.07–.78), .02	.29 (.09–.97), .04	138 (72–216)	128 (107–205)	11 (12%)	0
Cause of death								
Septicemia/meningitis^g^	36 (20%)	14 (36%)	1.06 (.57–1.97), .84	1.50 (.78–2.86), .22	76 (39–136)	79 (51–126)	20 (22%)	9 (45%)
Unknown cause^h^	33 (18%)	3 (8%)	.25 (.08–.81), .02	.35 (.10–1.16), .09	117 (47–207)	128 (107–205)	14 (16%)	0
Extrapulmonary cryptococcosis	20^i^ (11%)	0	…	…	50 (28–109)	…	14 (16%)	0
Other non-WHO stage 4 brain disease	16^j^ (9%)	0	…	…	88 (56–188)	…	8 (9%)	0
Tuberculosis	14 (8%)	1 (3%)	.19 (.03–1.48), .11	.29 (.04–2.32), .25	72 (36–143)	…	8 (9%)	0
Pulmonary	9 (5%)	0					*6* (7%)	0
Extrapulmonary	5 (3%)	1 (3%)					*2* (2%)	0
Pneumonia^g^	10 (6%)	11 (28%)	3.01 (1.28–7.09), .01	4.72 (1.91–11.7), .001	34 (28–274)	41 (29–138)	6 (7%)	8 (40%)
Other WHO 4 OIs (toxoplasmosis, PCP, CMV, cryptosporidiosis, isosporiasis)	8 (4%)	1 (3%)	.34 (.04–2.74), .31	.67 (.08–5.66), .72	49 (40–88)	…	6 (7%)	1 (5%)
Wasting, diarrhea, gastrointestinal	6 (3%)	3 (8%)	1.36 (.34–5.45), .66	2.83 (.68–11.8), .15	171 (69–283)	100 (39–107)	3 (3%)	1 (5%)
AIDS-defining malignancy (KS, lymphoma)	6 (3%)	0	…	…	179 (119–206)	…	0	0
Anemia, neutropenia, thrombocytopenia without sepsis	6 (3%)	0	…	…	78 (59–102)	…	3 (3%)	0
Hepatic	6 (3%)	0	…	…	212 (75–339)	…	2 (2%)	0
Trauma, obstetric, suicide	6 (3%)	1 (3%)	.45 (.05–3.76), .46	.30 (.04–2.48), .26	195 (181–216)	…	1 (1%)	0
Other lung disease	3^k^ (2%)	0	…	…	95 (81–141)	…	1 (1%)	0
Malaria	2 (1%)	1 (3%)	…	…	…	…	1 (1%)	0
Renal (non-AIDS)	2^l^ (1%)	0	…	…	…	…	0	0
Cerebrovascular disease	0	3 (8%)	…	…	…	137 (17–186)	0	1 (3%)
Other single causes in either trial	5^m^ (2%)	1^n^ (3%)	…	…	…	…	3 (3%)	0

Abbreviations: ART, antiretroviral therapy; ARROW, Antiretroviral Research for Watoto; CI, confidence interval; CMV, cytomegalovirus; DART, Development of Antiretroviral Therapy in Africa; HIV, human immunodeficiency virus; HR, hazard ratio; IQR, interquartile range; KS, Kaposi sarcoma; OI, opportunistic infection; PCP, pneumocystis pneumonia; WHO, World Health Organization.

^a^ Adjusted for CD4/CD4% categories (0–49 cells/μL, 0%–4%; 50–99 cells/μL, 5–9%; ≥100 cells/μL, ≥10%). Sub–hazard ratios corresponding to the cumulative incidence [26] were similar (data not shown).

^b^ For groups with 3 or more deaths.

^c^ Eight primarily ART related: 7 zidovudine (1 alone, 2 + cotrimoxazole, 2 + tenofovir, 1 + sulphadiazine/pyrimethamine, 1 + sulphamethoxazole), 1 nevirapine; 2 primarily other medication related only: 1 rifampicin/isoniazid/ethambutol/pyrazinamide, 1 dapsone.

^d^ Primarily ART related: zidovudine + cloxacillin + ceftriaxone.

^e^ Twenty-four uncertain whether primarily ART related (or HIV related): 21 zidovudine (12 alone, 3 + cotrimoxazole, 1 + tenofovir, 1 + tenofovir + amoxicillin + paracetamol, 1 + tenofovir + ciprofloxacin + diclofenac, 1 + tenofovir + dexamethasone + carbamazepine, 1 + tenofovir + rifampicin + isoniazid, 1 + tenofovir + rifampicin + isoniazid + ethambutol + pyrazinamide + ceftriaxone), 2 nevirapine, 1 stavudine; 1 uncertain whether primarily other medication related (or HIV related): 1 fluconazole.

^f^ Four uncertain whether primarily ART related (or HIV related): 4 zidovudine (2 alone, 2 + cotrimoxazole); 1 uncertain whether primarily other medication related (or HIV related): cotrimoxazole.

^g^ Organisms isolated from bacterial infections (blood cultures unless stated): DART: *Streptococcus pneumoniae* (3), *Escherichia coli* (4, plus 1 with urinary *E. coli* only), *Staphylococcus aureus* (2). ARROW: *S. pneumoniae* (3), *S. aureus* (1), *Pseudomonas aeruginosa* (1), *Klebsiella pneumoniae *+ *Enterococcus* spp (1), plus 1 with urinary *K. pneumoniae* only and 1 with *Salmonella* spp isolated from stool.

^h^ Primary cause of death could not be determined (eg, because the patient died at home or presented very sick without time for diagnostic tests).

^i^ Nineteen cryptococcal meningitis, 1 cryptococcemia.

^j^Participant fulfilled clinical criteria for at least 1 of cerebral toxoplasmosis, cryptococcal meningitis, tuberculosis meningitis, or progressive multifocal leukoencephalopathy, but no diagnostic tests were done and/or the patient failed to respond to first-line treatment and died without further investigations.

^k^2 pulmonary embolus; 1 chronic obstructive pulmonary disease.

^l^ Glomerulonephritis, chronic renal failure in a patient with type 1 diabetes and hypertension.

^m^ Stevens-Johnson syndrome, diabetes, lactic acidosis, non-AIDS cancer (carcinomatosis), cardiomyopathy.

^n^ HIV encephalopathy.

A large range of causes of death was observed in adults and children, the most common being septicemia/meningitis, with pneumonia also a common child cause of death (Table 2). Causes of death in the first 3 months were similar to the first year overall. Organisms were identified in relatively few cases but were typical for the setting, including *Streptococcus pneumoniae*, *Escherichia coli*, *Staphylococcus aureus*, and *Klebsiella pneumoniae*. Cryptococcus accounted for 11% of adult deaths, but no child deaths (exact *P* = .03 vs noncryptococcal cause); tuberculosis accounted for 8% vs 3% of deaths, respectively (adjusted *P* = .25). The only strong evidence for differing incidence of death between adults and children was from pneumonia (4.72-fold higher risk in children, *P* = .001; 3.73-fold higher risk pooling all respiratory-related deaths, *P* = .002). Uncertain or unlikely relationship to HIV/drugs and uncertain cause of death tended to be reported less frequently in children (adjusted *P* = .04, .08, and .09, respectively). Of causes with ≥10 adult deaths, the earliest were from pneumonia (median 34 days on ART), then cryptococcal disease (median 50 days), tuberculosis (median 72 days), septicemia/meningitis (median 76 days), other severe brain disease (median 88 days), and unknown causes (median 117 days). Child deaths from pneumonia and septicemia/meningitis occurred at similar timescales (median 41 and 79 days, respectively).

**Figure 3.**
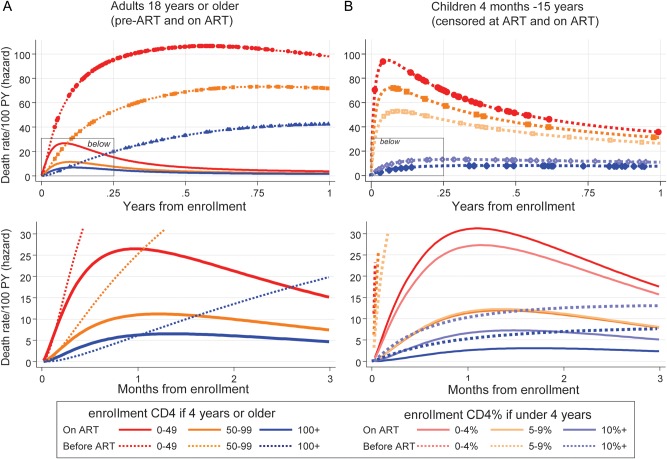
Daily risk of death and survival through 1 year before and on antiretroviral therapy (ART). Flexible parametric model [20, 21] on log-normal scale with 1 interior knot. Points show times when deaths occurred. Fewer than 40 children aged 1–3 years with 0%–4% pre-ART CD4 count were enrolled in the 3Cs4kids study (data not shown). Abbreviations: ART, antiretroviral therapy; PY, person-years.

One question is whether high early mortality risks merely reflected carryover effects of late presentation, with a delay in ART effectiveness in those with low pre-ART CD4 counts. We therefore estimated mortality risks over the first year after enrollment in adults with CD4 <200 cells/μL in the Entebbe cohort [28] and children in the 3Cs4kids cohort collaboration [29] who presented for care with low CD4 but did not receive ART, using the same CD4 strata and models as above (see Supplementary Methods for cohort details). Without ART, 40% (206/514) of adults and 9% (126/1377) of children died during the first year after enrollment; no adults, but 451 (33%) children, actually initiated ART during this first year and were censored. Figure 3 shows that high mortality risks were similar in the first 30 days after enrollment to early risks on ART. Without ART, risks remained high, particularly for adults, whereas those starting ART experienced rapid drops in risk.

## DISCUSSION

Although the substantial benefits of ART are clear, it remains uncertain how best to reduce excess early mortality in severely immunocompromised HIV-infected individuals initiating ART in low-income countries [2, 4]. Our main finding is that patterns of changing mortality risk in the first year on ART are indistinguishable in adults and children ≥4 years; both experience similarly high mortality risks in the first 3 months on ART if they have low pre-ART CD4 counts. Children aged <4 years with low CD4% are at similarly high risk for mortality. Nevertheless and importantly, risks on ART are lower than mortality risks without ART. While earlier HIV diagnosis and prompt ART initiation remain key goals, similarities between adults and children call for interventions to target reductions in early mortality in both; simplification and harmonization of management approaches across both groups would be an advantage in most resource-limited settings. Our findings contrast with the only comparison of early mortality in adults and children to date, which suggested somewhat lower overall risks over the first year on ART in 2- to 14-year-olds entering the Malawi national ART program [15]; however, data on CD4, which is the key determinant of early mortality, were not available.

In addition to comparing mortality risks in adults and children, we also examined potential mechanisms for increased early mortality to inform future interventions. Possible reasons for an early limited increase in mortality risk after ART initiation include ART toxicity, immune reconstitution inflammatory syndrome (IRIS), or time required for ART to become effective. Our study does not support a major role of ART toxicity; although a small number of deaths were adjudicated as primarily drug-related (mainly to zidovudine [anemia/neutropenia/sepsis] and nevirapine [hepatic failure]), participants initiating ART with low CD4 counts suffered disproportionately from both drug- and HIV-related deaths, suggesting that advanced immunodeficiency at ART initiation, rather than ART alone, played a role in toxicity-related deaths [30]. We did not directly assess the contribution of IRIS because these clinical criteria were not systematically recorded in 2003–2004, when the early deaths occurred in DART. It is pertinent that, even if IRIS was a mechanism, early mortality risks on ART were no greater than observed in similar groups without ART. This supports recent trial findings that, despite increased IRIS, early ART significantly reduced mortality in patients with advanced HIV disease and tuberculosis [31, 32]. The fact that mortality risks in the first weeks on ART were similar to those without ART might also suggest that pre-ART risks persist until ART reaches maximal effectiveness. If this were the case, then increasing ART potency (eg, by adding an integrase inhibitor to initial ART) might benefit patients with advanced immunodeficiency. Integrase inhibitors are obvious candidates for such induction-maintenance strategies as they achieve the most rapid viral load declines [33].

The diverse early causes of death observed in adults and children present a challenge in selecting interventions targeting specific causes. Although certain opportunistic infections (cryptococcus, tuberculosis) made clear contributions, individually their effects were modest, similar to findings from other studies in adults [34]. Although no child died from cryptococcal disease, this is probably owing to low numbers, as deaths have been reported in older children [35–37]. Our findings suggest that any augmented prophylaxis approach to reduce early morbidity/mortality needs to cover multiple organisms. Although fluconazole [38], isoniazid [39], and cotrimoxazole [40, 41] prophylaxes have important benefits, it remains unclear whether toxicity risks associated with simultaneous initiation with 3-drug (or 4-drug) ART would outweigh any potential advantages in patients presenting with very low CD4 counts. Aside from toxicity concerns, the considerably increased pill burden and potential for drug–drug interactions suggests that such strategies need formal evaluation.

Invasive bacterial infections (septicemia/meningitis/pneumonia) were the commonest early cause of death in adults and children. Similarly high contributions of bacterial infections to early mortality on ART were described in a comparison of adult cohorts in Brazil and the United States [42]. In contrast to tuberculosis and cryptococcal disease, little attention has been paid to this finding in the wider literature, although septicemia and pneumonia were relatively commonly reported as “other” cause of death in a recent meta-analysis of 1-year mortality in adults [34] and the importance of bacterial infections in child mortality has been noted [43]. Cotrimoxazole prophylaxis, received by all ARROW children and 62% DART adults at ART initiation, has major mortality benefits in HIV-infected persons [40, 41], presumed to occur through reduction of bacterial infections. Given high rates of cotrimoxaozole resistance, our data suggest that additional broad-spectrum antibiotic prophylaxis might also improve outcomes among adults and children presenting with low CD4 counts.

As previously reported [12, 34, 44], low BMI and hemoglobin were associated with higher early mortality in adults and children in our study. Of note, BMI associations were present even with mildly abnormal BMI, suggesting a potential role for additional nutritional supplementation in reducing early mortality following ART initiation [45]. This approach could improve drug absorption, known to be impaired in severe HIV disease, and possibly adherence, as many patients report feeling acute hunger after starting ART [46], probably reflecting a profound catabolic state induced by severe HIV infection, which abruptly reverses with ART. However, moderately sized trials in HIV-infected adults [47] and in patients with tuberculosis [48] starting treatment with severe malnutrition (BMI <18.5) have shown no significant effect of ready-to-eat and fortified soya foods on mortality, although early weight, BMI, and CD4 gains were observed. Therefore, the role of supplementation in reducing early mortality in those without severe malnutrition remains unclear.

Our trial data have 3 major advantages over previous studies for investigating early mortality. First, vital status was accurately and completely ascertained in contrast to most programs [27, 34], thus limiting the impact of mortality misclassification. Even the assumption that all those lost to follow-up in the first year had died (1.1% adults, 0.8% children) did not alter results. Second, causes of death were assigned by an independent committee, based on a structured narrative and without knowledge of the randomized group or CD4 (part of the randomizations). Third, we used innovative flexible parametric models to directly assess how mortality risk changed over time on ART. The alternative strategy, categorizing time on ART [2, 10–13, 15, 43, 49, 50], produces risk estimates that change abruptly, making them biologically implausible [14], and may poorly represent the underlying data. In recognition of this problem, 2 recent papers used a piecewise Weibull model (which nevertheless makes strong assumptions about changing risks) [9] or smoothed hazards from semi-parametric Cox models [51].

WHO guidelines advocate ART initiation in HIV-infected adults and children aged ≥5 years at a CD4 count of <350 cells/μL [6], but substantial numbers of individuals continue to present for care late in HIV infection [7, 52–54]. Given experiences in high-income countries [55, 56], late presentation will continue in ART programs for the foreseeable future. However, the consequences of such late presentation, in terms of early morbidity/mortality on ART, are far more severe in low-income settings. To maximize ART benefits, it is essential to identify which, if any, interventions could reduce high early death rates if given with ART. Recent experiences in a trial of hydroxychloroquine, which significantly increased HIV viral load in ART-naive patients despite being expected to reduce immune activation [57], demonstrate the importance of testing potential interventions in randomized controlled trials. Our findings do not identify any single plausible mechanism; rather, they suggest that anti-HIV, anti-infection (or enhanced prophylaxis), and anti-malnutrition/malabsorption may all be important potential approaches. The REALITY trial (ISRCTN43622374) plans to address these questions in adults and older children in a 2 × 2 × 2 factorial design from 2012. Our findings also demonstrate strong similarities in early mortality patterns between adults and children. Where the same health providers treat both age groups, as in most of sub-Saharan Africa, integrating research will likely provide the most relevant evidence base for future management.

## Supplementary Data

Supplementary materials are available at *Clinical Infectious Diseases* online (http://www.oxfordjournals.org/our_journals/cid/). Supplementary materials consist of data provided by the author that are published to benefit the reader. The posted materials are not copyedited. The contents of all supplementary data are the sole responsibility of the authors. Questions or messages regarding errors should be addressed to the author.

Supplementary Data
