# Supplementary material for: Mortality in the Year Following Antiretroviral Therapy Initiation in HIV-Infected Adults and Children in Uganda and Zimbabwe
Source: Clin Infect Dis. 2012 Sep 12;55(12):1707–18. doi: 10.1093/cid/cis797 (PMC3501336; doi:10.1093/cid/cis797)
Supplement: Supplementary Data [file supp_55_12_1707__index.html]

MORTALITY IN THE YEAR FOLLOWING ANTIRETROVIRAL THERAPY INITIATION IN HIV-INFECTED ADULTS AND CHILDREN IN UGANDA AND ZIMBABWE — Mortality in the Year Following Antiretroviral Therapy Initiation in HIV-Infected Adults and Children in Uganda and Zimbabwe — Mortality in the Year Following Antiretroviral Therapy Initiation in HIV-Infected Adults and Children in Uganda and Zimbabwe — Supplementary Data 

# Mortality in the Year Following Antiretroviral Therapy Initiation in HIV-Infected Adults and Children in Uganda and Zimbabwe

## Supplementary Data

Supplementary Data

**Files in this Data Supplement:**

- Supplementary Data - Doc file
