# Supplementary material for: Mortality in the Year Following Antiretroviral Therapy Initiation in HIV-Infected Adults and Children in Uganda and Zimbabwe
Source: Clin Infect Dis. 2012 Sep 12;55(12):1707–18. doi: 10.1093/cid/cis797 (PMC3501336; doi:10.1093/cid/cis797)
Supplement: Supplementary Data [file supp_cis797_cis797supp.doc]

**ONLINE ONLY MATERIAL**

eMETHODS

eTable 1 Multivariable model for mortality in the first year on ART in adults and children

**eMETHODS**

**Details of statistical model**

Flexible parametric survival models are based on the standard Weibull, log-normal or proportional odds models. The underlying models have monotonic (ie always increasing or always decreasing) hazards, but the flexible parametric models introduce additional terms in the hazard linearization (via natural cubic splines) which allow the death rate to increase and then decrease or vice versa. The Akaike Information Criterion (AIC) was used to identify the best fitting underlying hazard model (Weibull, log-normal, odds) and the number of interior knots for the natural cubic splines (between 1 and 6)[^1^](#_ENREF_1), separately in adults (DART) and children (ARROW). As numerous studies have identified pre-ART CD4 as the most important predictor of mortality on ART, AIC-based selection of the underlying model was performed adjusted for pre-ART CD4 as an explanatory factor, and also allowing the variation in death rates over time to differ according to pre-ART CD4 (stratified model). In both DART and ARROW, the best fitting model according to AIC was log-normal with 1 interior knot at the 50th percentile of the uncensored survival times, plus 2 boundary knots at their minimum and maximum, and pre-ART CD4 included as an explanatory variable rather than a stratification factor on the baseline hazard.

The same modelling strategy was used to identify the best flexible parametric model to describe risk of death in adults and children presented for care with low CD4 but not receiving ART.

**Details of pre-ART cohorts used to compare variation in the risk of death over time on ART with mortality risks in those not not receiving ART**

We observed greatest mortality risks during the first 30-50 days on ART, risks which then subsequently declined substantially. One possible explanation is that these high initial risks are a “carry-over” from high risks in adults/children with low CD4s not receiving ART. To investigate this, we fitted the same flexible parametric models described above to time from cohort enrolment to the earliest of death, lost to follow-up or 1 year in pre-ART adult and paediatric cohorts, in order to compare how mortality risks changed over time in those off ART with those on ART, and whether early risks on ART were still substantially smaller, similar to, or even greater than those off ART. (The last scenario would, for example, support a role for increased mortality risk as a consequence of immune reconstitution inflammatory syndrome (IRIS).) We used cohorts which took individual consent rather than national programmes to provide at least partial matching for the consent required to join the DART/ARROW trials, and because these cohorts undertook at least some defaulter tracing. We also excluded other cohort participants who were known to not meet the respective trial’s entry criteria (eg based on enrolment CD4). For adults, the Entebbe cohort is a population-based cohort in one of the Ugandan DART trial sites enrolling HIV-infected adults aged 15–59 years in WHO clinical stage 1-3. Participants had 6-monthly follow-up with active defaulter tracing to ascertain vital status. Analysis included 514 adults 18-59 years with CD4 <200 cells/mm^3^ at enrolment (to match DART criteria) who were enrolled between October 1995-June 1998 (5 years before ART first became available, and >2 years before cotrimoxazole prophylaxis evaluation started[^2^](#_ENREF_2)). For children, the 3Cs4kids collaboration includes longitudinal data from 10 studies actively following children pre-ART (9 Africa, 1 Brazil)[^3^](#_ENREF_3). Analysis included 1377 children enrolled between 1990-2005 aged 1 -15 years with a CD4 count at enrolment. As a number of children subsequently initiated ART in these cohorts, pediatric data were also censored at ART initiation. Despite active follow-up, a larger proportion was lost before 1 year in these pre-ART studies (55(11%) adults, 213(15%) children). Analyses censoring those lost may therefore underestimate mortality risk if those lost are actually more likely to have died, and are thus conservative.

**References**

1. Lambert PC, Royston P. Further devlopments of flexible parametric models for survival analysis. *The Stata Journal.* 2009;9:265-290.

2. Watera C, Todd J, Muwonge R, et al. Feasibility and effectiveness of cotrimoxazole prophylaxis for HIV-1-infected adults attending an HIV/AIDS clinic in Uganda. *J Acquir Immune Defic Syndr.* Jul 2006;42(3):373-378.

3. 3Cs4kids Cohort Collaboration. Markers for predicting mortality in untreated HIV-infected children in resource-limited settings: a meta-analysis. *AIDS.* Jan 2 2008;22(1):97-105.

**eTable 1 Multivariable model for mortality in the first year on ART in adults and children**

| Factor at ART initiation | Multivariable model 1*  AF** (95% CI) p | Multivariable model 2  AF** (95% CI) p |
| --- | --- | --- |
| Age: per 10 years older | 0.99 (0.95-1.04) 0.83 | 1.07 (1.01-1.13) 0.02 |
| Sex: female vs male | 0.91 (0.80-1.04) 0.16 | 0.96 (0.83-1.11) 0.56 |
| Centre: B vs A  C vs A  D vs A | 1.00 (0.84-1.19) 0.65  0.99 (0.82-1.18)  1.12 (0.90-1.40) | 1.13 (0.94-1.36) 0.08  1.05 (0.86-1.27)  1.33 (1.05-1.67) |
| CD4 group:  50-99 vs 0-49†  ≥100 vs 0-49‡ | 0.71 (0.60-0.85) <0.0001  0.58 (0.50-0.68) | 0.75 (0.63-0.90) <0.0001  0.63 (0.53-0.73) |
| WHO stage: 3 vs 2  4 vs 2 | 1.45 (1.17-1.79) <0.0001  1.89 (1.50-2.36) | 1.33 (1.06-1.67) 0.0005  1.61 (1.26-2.05) |
| On cotrimoxazole/dapsone | 0.76 (0.65-0.88) 0.0003 | 0.76 (0.65-0.90) 0.001 |
| Haemoglobin: per g/dl higher | - | 0.91 (0.87-0.95) <0.0001 |
| BMI z-score (WHO): per unit higher  below 0  above 0 | - | 0.78 (0.73-0.84) <0.0001  1.00 (0.87-1.16) |

* not including effects of pre-ART BMI and haemoglobin, which might be expected to vary more between adults and children.

** AF=Acceleration factor from a log-normal model (the best fitting flexible parametric model), the amount by which time to death is increased per unit higher/relative to a reference category

† or 5-9% vs 0-4% in those under 4 years

‡ ≥10% vs 0-4% in those under 4 years

Note: no evidence of interactions with pre-ART CD4 group in either model (p>0.3).
